# Norepinephrine formulation for equivalent vasopressive score

**DOI:** 10.1186/s13054-023-04354-4

**Published:** 2023-02-16

**Authors:** Nicolas Mongardon, Quentin de Roux, Marc Leone, Philippe Guerci

**Affiliations:** 1 Service d’Anesthésie-Réanimation Chirurgicale, DMU CARE, Assistance Publique-Hôpitaux de Paris (AP-HP), Hôpitaux Universitaires Henri Mondor, 1 Rue Gustave Eiffel, 94010 Créteil, France; 2 Université Paris Est Créteil, Faculté de Santé, 94010 Créteil, France; 3 U955-IMRB, Equipe 03 “Pharmacologie et Technologies pour les Maladies Cardiovasculaires (PROTECT)”, Inserm, Univ Paris Est Créteil (UPEC), Ecole Nationale Vétérinaire d’Alfort (EnvA), 94700 Maisons-Alfort, France; 4 Service d’Anesthésie et de Réanimation, Assistance Publique-Hôpitaux Universitaires de Marseille, Aix Marseille Université, Hôpital Nord, 13015 Marseille, France; 5 Department of Anesthesiology and Critical Care Medicine, Institut Lorrain du Coeur et des Vaisseaux, University Hospital of Nancy, 57000 Vandoeuvre-Les Nancy, France; 6 INSERM U1116, DCAC, University of Lorraine, Nancy, France

## Correspondence

Kotani and colleagues should be commended for comparing the vasopressive effects of most vasopressors available on the market. Their work aims at harmonizing the vasopressive burden in patients with shock [1].

In our opinion, the authors forgot to mention that, depending on countries and institutions, two formulations of norepinephrine are available: either as tartrate/bitartrate at 2 mg/mL or as base at 1 mg/mL. This means that the equipotent vasopressive formulations can require to adapt the dose by a factor 2, a conversion factor of paramount importance in daily practice. The Surviving Sepsis Campaign (SCC) 2021 recommended adding vasopressin in septic shock patients when the dose of norepinephrine reaches 0.25 −0.5 μg/kg/min [2]. For an 80-kg adult, this translates by introducing vasopressin at a threshold ranging from 1.2 mg/h of norepinephrine when the base formulation is used at the lowest recommendation to 4.8 mg/h when the tartrate/bitartrate formulation is used at the higher recommendation. Interestingly, in an international survey on the adhesion to SSC guidelines, 50% of 820 respondents were unaware of the formulations used in their units [3]. This bias can lead to unwanted high doses of norepinephrine and misinterpretation of clinical trial results. In addition, delayed vasopressin introduction may be less effective than an early multimodal strategy based on combination of several vasopressors [4, 5].

In conclusion, there is a need for homogenizing the norepinephrine formulation in order to improve the interprofessional communication on vasopressive equivalence scores across the different publications and practices all over the world.

## Data Availability

Not applicable.
